# Frame overlap Bragg edge imaging

**DOI:** 10.1038/s41598-020-71705-4

**Published:** 2020-09-10

**Authors:** Matteo Busi, Jan Čapek, Efthymios Polatidis, Jan Hovind, Pierre Boillat, Anton S. Tremsin, Winfried Kockelmann, Markus Strobl

**Affiliations:** 1 Paul Scherrer Institute, Laboratory for Neutron Scattering and Imaging, Forschungsstrasse 111, 5232 Villigen, Switzerland; 2 University of California, Berkeley, CA 94720 USA; 3 ISIS Facility, STFC-Rutherford Appleton Laboratory, Chilton, OX11 0QX UK

**Keywords:** Imaging techniques, Materials science

## Abstract

Neutron Bragg edge imaging enables spatially resolved studies of crystalline features through the exploitation and analysis of Bragg edges in the transmission spectra recorded in each pixel of an imaging detector. Studies with high spectral resolutions, as is required e.g. for high-resolution strain mapping, and with large wavelength ranges have been largely reserved to pulsed neutron sources. This is due to the fact, that the efficiency for high wavelength resolution measurements is significantly higher at short pulse sources. At continuous sources a large fraction of the available neutrons must be sacrificed in order to achieve high wavelength resolution for a relevant bandwidth e.g. through a chopper system. Here we introduce a pulse overlap transmission imaging technique, which is suited to increase the available flux of high wavelength resolution time-of-flight neutron Bragg edge imaging at continuous neutron sources about an order of magnitude. Proof-of-principle measurements utilizing a chopper with a fourfold repeated random slit distribution of eight slits were performed at a thermal neutron beamline. It is demonstrated, that disentanglement of the overlapping pulses is achieved with the correlation theorem for signal processing. Thus, the Bragg edge pattern can be reconstructed from the strongly overlapping Bragg edge spectra recorded and the results demonstrate the feasibility of the technique.

## Introduction

Neutron Bragg edge imaging^1^ has proven to be a powerful tool for the non-destructive characterization of materials with regards to spatially resolved assessment of crystalline characteristics such as crystalline phase distributions^2–5^, strain fields^6–8^ and texture features^9,10^. In contrast to X-rays, neutrons are able to probe the volume of bulk engineering materials and in contrast to neutron diffraction, Bragg edge imaging can provide superior spatial resolution. Bragg edges in the transmission spectrum are the signature of Bragg scattering from polycrystalline materials. For a specific crystal lattice family *hkl*, with lattice spacing $d_{\textit{hkl}}$, the scattering angle increases with the wavelength ($\lambda$) up to $\lambda = 2 d_{\textit{hkl}} \sin {(\pi /2)}$. Beyond this wavelength, the Bragg condition cannot be satisfied any longer, which results in a sharp drop of the material’s attenuation, the so-called Bragg edge. Thus, the analysis of the Bragg edges allows for the characterization of crystalline features of materials such as lattice strains or phase fractions^1^.

Bragg edge imaging can be realized in multiple ways at both pulsed and continuous neutron sources, with different trade-offs between flux and wavelength resolution. Applications at continuous sources are dominated by monochromatization techniques using crystal monochromators, in particular double crystal monochromators^10^, and velocity selectors^11^. This implies rejecting the largest fraction of the available neutron flux. Likewise, recording a wavelength dependent attenuation spectrum in this case implies to scan through wavelengths measuring images for consecutive wavelengths one after the other. While for some applications only a few wavelengths need to be probed and the wavelength resolution of velocity selectors ($\delta \lambda /\lambda \approx 10\%$) or conventional crystal monochromators ($\delta \lambda /\lambda$ of a few $\%$) is sufficient^3,4,10^, for several applications a higher wavelength resolution ($\delta \lambda /\lambda \le 1\%$) is required and probing a significant wavelength range of a few Ångströms quasi-simultaneously is advantageous. In the latter case, which includes key applications such as strain mapping^6,7,12,13^, time resolved investigations such as phase transformation studies^14,15^ but also multi-phase identification and mapping, a Time-of-Flight (ToF) approach is the method of choice. Although ToF imaging at continuous sources has been demonstrated using chopper systems^7,16^, high-resolution broadband measurements are realized more efficiently at pulsed spallation sources. At the latter, no additional flux penalty is implied in achieving the required time structure of the beam. Thus, the Fig. of Merit (FOM) of sources with respect to wavelength dispersive measurements, such as Bragg edge imaging, scales with the peak brightness of the source, rather than with time-averaged neutron flux^17^.

**Figure 1:**
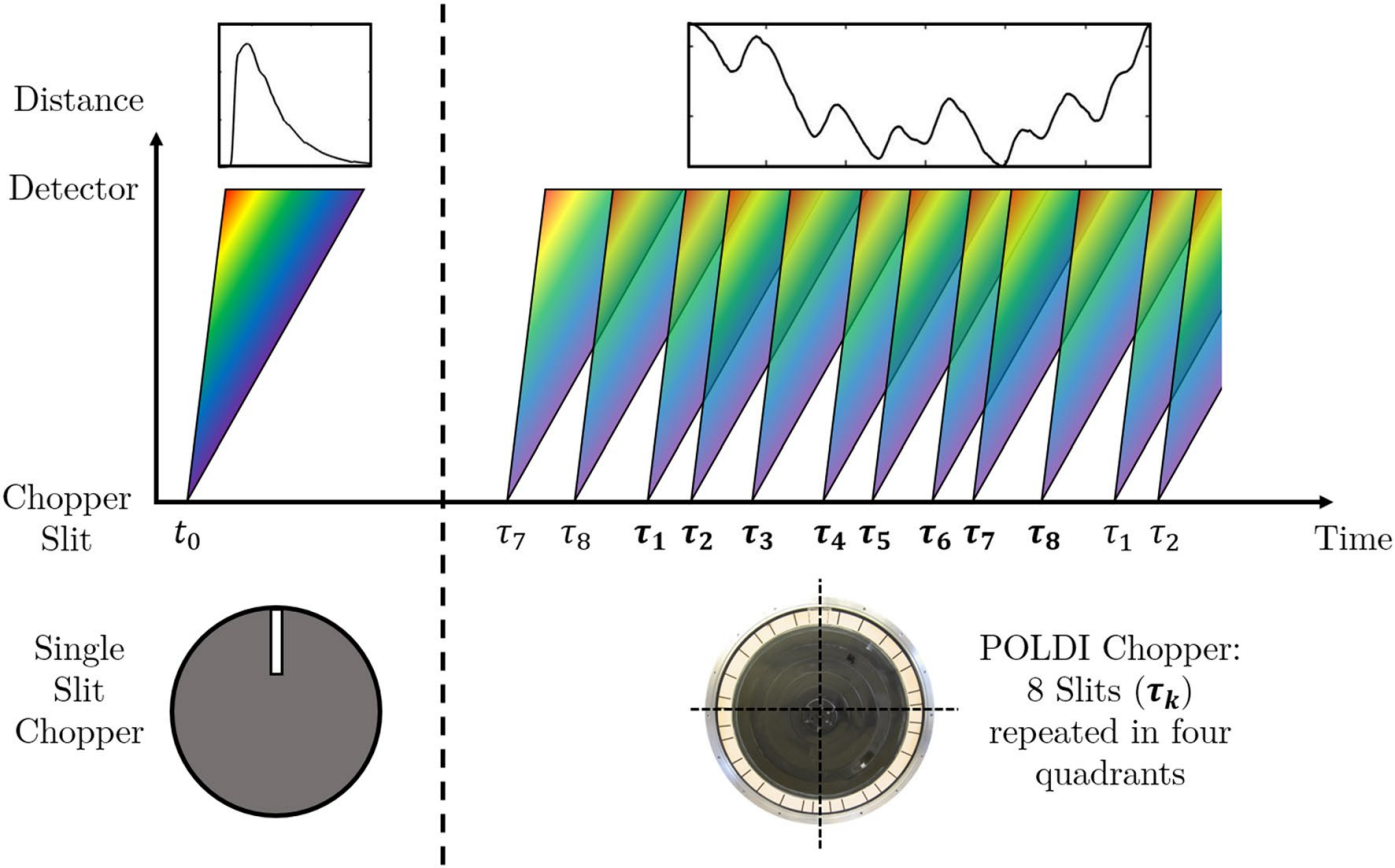
Sketch of the conventional Bragg edge imaging and the proposed Frame Overlap Bragg edge Imaging techniques carried out in ToF mode using chopper systems. On the left side, using a conventional single slit chopper, single neutron pulses are separated and the measured ToF can be directly converted to a neutron wavelength by knowing the distance between the chopper and the detector. On the right side, using a multiple slit chopper^22^ (POLDI chopper), neutrons having different wavelengths may arrive at the detector with the same ToF. The overlap of the spectra does not allow the direct conversion to wavelength and obscures the identification of Bragg edges. The goal of the FOBI technique is to disentangle the overlap between different pulses, assuming the times at which they are emitted are known.

In this work, we present a novel approach for ToF Bragg edge transmission imaging for continuous neutron sources, which is suited to increase the FOM for such studies at these sources by about an order of magnitude. The method, named Frame Overlap Bragg edge Imaging (FOBI), employs a chopper system at a continuous neutron source that allows for manifold pulse overlap, in contrast to conventional ToF Bragg edge imaging where the individual pulses are separated (Fig. 1). The approach is inspired by coded source imaging^18–21^, which is translated into the ToF domain using a chopper with multiple slits distributed in a repeated pseudorandom pattern, each of which is the source of a well-defined short neutron beam pulse (Fig. 1). In this way, the flux reduction caused by the chopper is reduced dramatically by correspondingly increasing the duty cycle. However, due to the superimposition in ToF of neutrons having different wavelengths the straightforward detection and analysis of Bragg edges are obscured. In this work, we provide a formal description of the method for the retrieval of absolute wavelength spectra in analogy to methods for imaging techniques using coded sources and we present proof-of-principle results from simulations and experiments.

## Method

In the FOBI approach, the measured signal at the detector is the superimposition of multiple neutron pulses having a ToF, *t*, distribution defined by the source spectrum. The resulting overlapped spectrum is as the correlation operation ($\star$) between the single pulse spectrum, $I_0(t)$, and the time structure function $\Delta (t)$. The last term represents the time delay of the consecutive pulses and it is approximated by multiple Dirac delta functions for the short chopper openings with time delays, $\tau _0^k$, according to the radial position of the slits $k=1$ to *K* on the chopper disk and rotation frequency *f*, and can be written as

1 $$\begin{aligned} \Delta (t) = \sum _k a_k \delta (t-\tau _0^k). \end{aligned}$$

Note that in the discretization of the Dirac’s delta into bins with defined ToF width, each term $\left[ a_k \delta (t-\tau _0^k)\right]$ is split between the two nearest adjacent bins, each with a proper weight corresponding to the distance of the bin from the time delay. The coefficients $a_k$ are added as a product to the Dirac’s deltas, in case the slits have different aperture sizes hence, different pulses. In this work, the slits have equal apertures and the coefficients were set to $a_k = 1$, assuming no source flux fluctuations in time.

In Bragg edge transmission imaging in particular the wavelength dependent coherent elastic cross section, $\sigma _\text {coh,el}$ (barns) is measured where it dominates the total cross section $\sigma =\sigma _\text {a}+\sigma _\text {s}$ in the linear attenuation coefficient, $\mu = N \sigma (\text {cm}^{-1})$. $\sigma _\text {a}$ and $\sigma _\text {s}$ are the absorption and scattering cross sections. *N* is the number density of the corresponding nuclei. The wavelength dependence of the cross sections implies a wavelength dependent linear attenuation coefficient $\mu (\lambda )$ and, thus, a wavelength dependent Beer-Lambert law to be applicable as:

2 $$\begin{aligned} T(\lambda ) = \frac{I(\lambda )}{I_0(\lambda )}=e^{-l \mu (\lambda )}, \end{aligned}$$

where $I_0(\lambda )$ and $I(\lambda )$ are the incident and transmitted neutron beam intensities with respect to transmission of a material with thickness *l*. In conventional ToF imaging, the conversion from time-of-flight *t* (s) to wavelength, $\lambda$ ($\AA$), is only dependent on the distance, $\text {L}$ (m), between the pulsed source and the detector, and the measured neutron flight time *t* through:

3 $$\begin{aligned} \lambda = \frac{ht}{\text {L}m} \approx 3957 \frac{t}{\text {L}} , \end{aligned}$$

where *h* is the Planck constant and *m* is the mass of the neutron. However, in the FOBI approach the actual time-of-flight, *t*, of detected neutrons is obscured through multiple possible burst times $\tau _0^k$. Therefore, prior to data analysis the overlap of pulses must be disentangled with the aim to retrieve the actual spectra of $I_0(\lambda )$ and $I(\lambda )$.

We start by expressing the open beam measurement $I_0^K$ as the correlation between the beam spectrum and the time structure function of the chopper pulses:

4 $$\begin{aligned} I_0^K (\tilde{t}) = \sum _k I_0 (t-\tau _k) = I_0 (t) \star \left[ \sum _k a_k \delta (t-\tau _k) \right] = (I_0 \star \Delta ) (t). \end{aligned}$$

In the notation, $\tilde{t}$, refers to the overlapped ToF domain, which is the directly measured one, whereas *t* refers to the actual neutron ToF. According to the correlation theorem^23^, we can then express the Fourier transform of the measured signal as the product of the Hermitian Fourier transform of the beam spectrum $I_0(t)$, with *t* according to Eq. (3) the ToF at a distance $\text {L}$ from a pulse source, and the Fourier transform of the time structure function $\Delta (t)$:

5 $$\begin{aligned} \mathscr {F}\{I_0^K(\tilde{t})\} = \left( \mathscr {F}^\dagger \{I_0\}\mathscr {F}\{\Delta \}\right) (t). \end{aligned}$$

Equation 5 can be inverted and rearranged to retrieve the spectrum:

6 $$\begin{aligned} I_0(t) = \mathscr {F}^{-1}\Bigg \{\frac{\mathscr {F}\{I_0^K\} \mathscr {F}\{\Delta \}}{|\mathscr {F}\{\Delta \}|^2 + c}\Bigg \}, \end{aligned}$$

where the Wiener coefficient^24^, *c*, is introduced to cope with inverse Fourier transform issues caused by noisy components in the frequency domain.

In a similar way as the open beam spectrum $I_0$, the beam transmitted through a sample can be expressed as:

7 $$\begin{aligned} I^K (\tilde{t}) = I_0^K(\tilde{t}) T(\tilde{t}) = \sum _k I_0 (t-\tau _k) T (t-\tau _k) = I_0 (t) T(t) \star \left[ \sum _k a_k \delta (t-\tau _k) \right] = (I_0 T \star \Delta ) (t) , \end{aligned}$$

allowing the retrieval of the transmission function:

8 $$\begin{aligned} T(t) = \frac{1}{I_0(t)}\mathscr {F}^{-1}\Bigg \{\frac{\mathscr {F}\{I^K\} \mathscr {F}\{\Delta \}}{|\mathscr {F}\{\Delta \}|^2 + c}\Bigg \}, \end{aligned}$$

equivalently to Eq. (6). The ToF, i.e. wavelength range in which the open beam and the transmission spectra are retrieved is determined by the period of the pseudorandom source pulse distribution and the spectral intensity distribution of the utilized beam. Due to the transition into the frequency domain and back in Eqs. (6) and (8), respectively, the absolute ToF of neutrons in the retrieved spectrum is lost. This implies that in the reconstructed time window, consecutive spectral ranges are still superimposed. The weight by intensities in the spectrum, however, enables retrieval of correct spectral features within the according dominating wavelength range of maximum flux. The spectrum, however, should display a single peak distribution, within which the probed range can thus be tuned by the choice of parameters defining the period $P_\text {ToF}=(nf)^{-1}$, where *n* is the number of repetitions of the same pseudorandom slit pattern on a single chopper disk. Note, however, that changes of the frequency will influence the wavelength resolution. Therefore, while no trade of flux versus resolution is possible due to the fixed duty cycle, only trading between resolution and retrieved wavelength range is possible. Due to the loss of absolute times, the correct transformation from time to wavelength requires a calibration using a well-known reference Bragg edge spectrum, as is generally used in ToF transmission imaging.

## Measurements

### Instrumentation

The experiments were carried out at the POLDI diffractometer^22^ at the SINQ continuous neutron source of the Paul Scherrer Institute. POLDI is equipped with a chopper with eight slits distributed in a pseudorandom pattern, repeated on each quarter ($n=4$) of the disk (see Fig. 1). The eight slits composing the repeating pattern are distributed at [0, 9.4, 21.5, 37.0, 50.4, 56.7, 67.4, 75.4] degrees. While the maximum feasible chopper frequency is 15000 revolutions per minute (rpm) ($f = 250$ Hz), the experiments were performed with a chopper speed set to 2000 rpm corresponding to a period $P_\text {ToF}$ of 30 ms to cover a suitable wavelength range. Each of the 32 slits has a width of 4 mm and a height of 40 mm on a disc with 350 mm radius, equivalent to an aperture of 0.7 degree. This yields a duty cycle of the chopper of $5.8\%$. The distance from the chopper to the detector *L* was about 14 m, implying a wavelength resolution of 0.65$\%$ at 2.53 $\AA$. To validate the FOBI method proposed in this work we have carried out a reference measurement at the same beam line using a single slit chopper. The chopper for this measurement had a disc of only 200 mm radius with a single slit of 1.8 mm width (0.5 degree aperture) resulting in a duty cycle of 0.15$\%$. The chopper was operated at 25 Hz with a corresponding ToF wavelength resolution at $\text {L}= 14$ m of $0.6\%$ at $2.53 \AA$. The source and sample transmission spectra measured with this instrument setting were used as reference for FOM calculations. Moreover, the reference spectra were used in an initial feasibility test to simulate the FOBI measurements and validate the mathematical description of the technique. Finally, the same sample’s attenuation spectrum was measured at the IMAT^25^ beamline, which uses a pulsed neutron source, to benchmark the performance at different neutron sources.

**Figure 2:**
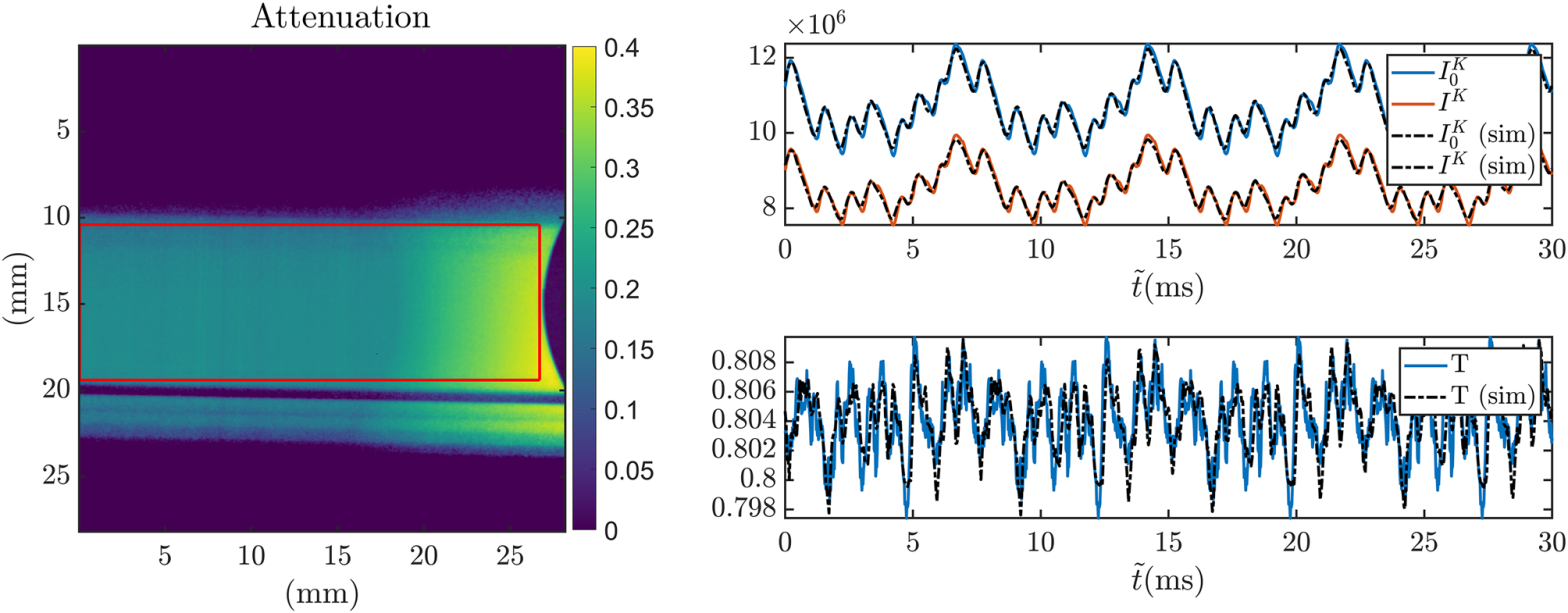
Left: Visualization of the attenuation of a sample (304 steel sheet, cruciform shape, after mechanical loading, compare sample section) in a ToF-integrated (full spectrum) image. The beam profile does not cover the entire detector’s field of view since the beam line is normally used as a diffractometer. The red box marks the Region of Interest (RoI) in which the data is merged and analyzed. Right: In the top frame, the blue and red solid lines are respectively the average incident and transmitted neutron signals within the marked RoI, as a function of the ToF bins. In the bottom frame the transmission ratio, T, is shown. The dashed black lines are the simulation counterparts using reference spectra.

The detector used for all the measurements was a Micro-Channel Plate (MCP)/Timepix^26^ detector for ToF imaging applications. The detector requires readout gaps in the data acquisition causing small gaps in the detected ToF spectra, which were recorded for periods corresponding to the chopper frequency. A standard correction algorithm is applied in order to correct for losses related to the detector’s limitation of detecting only one neutron per pixel per shutter^27^. Furthermore, five ToF bins adjacent to the readout gaps had to be truncated due to non-linear response artefacts not corrected by the algorithm. The detector is a flat panel 2D detector of $$512\times 512$$ pixels with a pixel size of 55 $$\mu \text {m}$$ for a field of view of $$28.16\times 28.16$$$$\hbox {mm}^2$$. Note that the beam of the POLDI beamline, which is typically used for diffraction experiments, does not cover the full detector area as it has a height with homogeneous intensity of approximately 10 mm (visible in Fig. 2).

### Samples

Several reference samples were measured. These include two dogbone samples produced by selective laser melting (SLM) of 304L steel (i.e. Fe-18Cr-8Ni) and a cruciform sample machined from conventional cold rolled 304 steel sheet. The cruciform sample had been subjected to a 90 degree load path change (90$^\circ$ LPC), while one of the SLM-produced dogbone samples was deformed up to 52$\%$ nominal uniaxial strain. Both samples exhibited deformation-induced martensitic phase transformation (FCC-to-BCC), and corresponding phase analyses had been performed before by diffraction, diffractive neutron imaging and EBSD, which is reported elsewhere^5,28^. One dogbone sample was studied in the as-built condition, containing only FCC austenite. Finally, a solid-oxide fuel cell anode material containing partially reduced NiO in a matrix of YSZ (Yttria-Stabilized Zirconia) was among the reference samples and had been investigated by diffractive neutron imaging producing phase maps^14^. Thus, all reference samples were well characterized with respect to Bragg edge neutron imaging. The corresponding features that can be analyzed via Bragg edge neutron imaging will be addressed in our FOBI data analyses in order to discuss the performance and demonstrate the potential of the method for efficient high-resolution Bragg edge imaging studies at continuous neutron sources.

## Results

### FOBI method

Figure 2 shows an attenuation contrast image of a sample based on the full spectrum, i.e. integrated over all ToF bins. Furthermore, it depicts the open beam ToF-data as well as the time-dependent transmission through the sample and their respective ratio, $T(\tilde{t})$, as measured with and calculated for the FOBI method. The FOBI data is measured with the POLDI chopper at 2000 rpm and the results of corresponding FOBI calculations are based on reference spectra measured at POLDI with the conventional chopper, in order to simulate FOBI results. It is observed, that a distinct signal shape is repeated four times in the presented data, corresponding to the four equal quadrants of the chopper with the same slit pattern measured during one chopper rotation period in accordance to the detection histogramming. Due to a moderate overlap, the eight relative maxima resulting from having eight overlapping source spectra are visible in the intensity signals. The difference in amplitude between the maxima is caused by the uneven distribution of the chopper windows. As expected, the transmission measurements appear very different from the typical sample transmission spectra due to the overlap of multiple shifted Bragg edge spectra. Nevertheless, the experiment matches almost perfectly the results of the calculated FOBI data. For the measured signal, the ToF domain in which the data is registered is discontinuous, due to the detector readout gaps. This is problematic for processing the Fourier and inverse Fourier transforms of Eqs. (6) and (8). Therefore, the four repeated patterns corresponding to the identical chopper quadrants are separated and added up to a single bandwidth ToF histogram. Because the readout gaps are not distributed evenly, they concern different ToF regions with respect to the four otherwise identical spectral repetitions. Consequently, the readout gaps could be filled in the merging procedure and a continuous function for retrieval was obtained. The resulting signal is repeated four times to restore the original time-of-flight domain.

**Figure 3:**
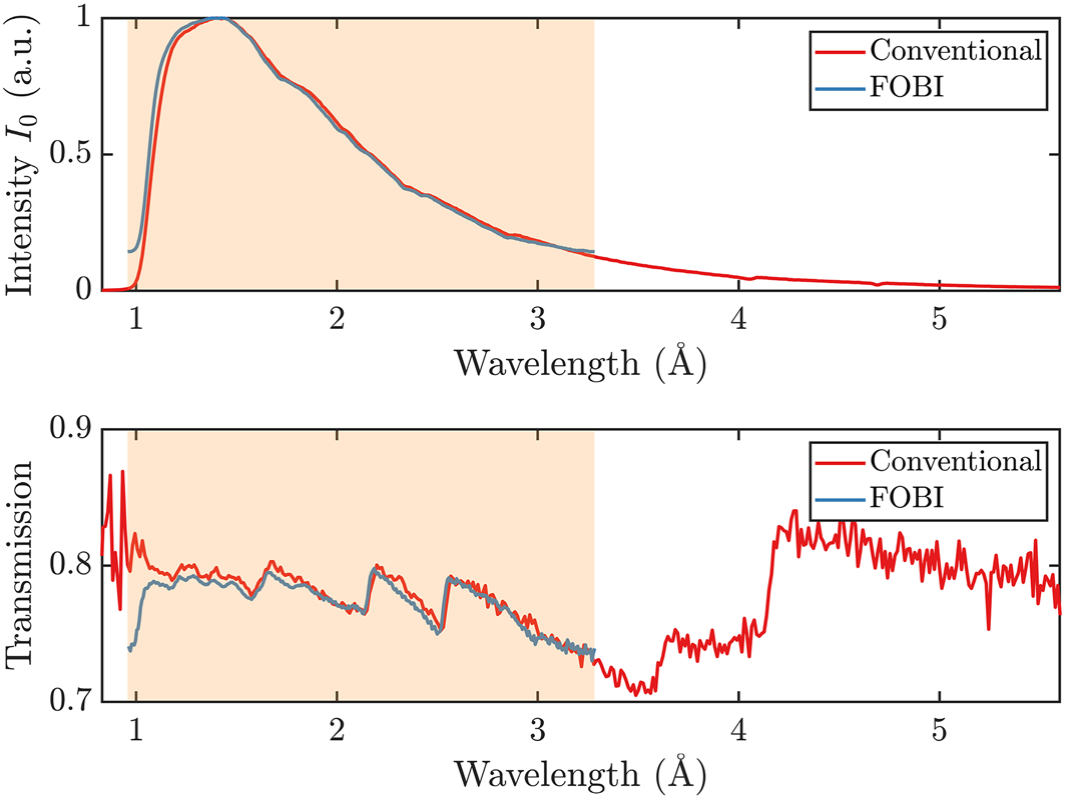
Top: The thermal neutron spectrum ($I_0$) of POLDI measured with the MCP detector with a conventional single slit chopper (red) and retrieved from the FOBI measurement (blue). The light-red shaded area marks the wavelength range that is well probed by the FOBI technique in our measurement, due to the chopper parameters and the specific POLDI spectrum. Bottom: Plots of the corresponding normalized sample transmission spectra for one of the steel samples.

Figure 3 shows the resulting neutron beam spectrum of POLDI and the normalized sample transmission spectrum retrieved by the FOBI method (Eqs. 6, 8), as well as the respective reference spectrum of POLDI measured with the conventional single slit chopper. The direct Fourier transform outputs the single pulse spectrum repeated four times, corresponding to the number of chopper’s slit pattern repetitions during one chopper period (detector trigger). These four repetitions have been summed into a single spectrum, which is reported in the figure. As discussed above, the retrieved spectra have an offset in the ToF domain that depends on the peak of the source spectrum and the bandwidth probed by the period of each pseudorandom chopper pattern. Therefore, for this work the calibration to find the offset and enable the conversion to wavelength was realized by fitting with the positions of the nominal Bragg edges at 2.16 $\AA$ and 2.53 $\AA$ of the cruciform sample. The wavelength band resulting from this calibration of the FOBI transmission spectra, which is highlighted in Fig. 3, ranges from 0.96 $\AA$ to 3.28 $\AA$ (light-red shaded area). The effect of the overlap is clearly visible in the low- and high-wavelength tails of the retrieved spectrum, i.e. the retrieved spectral regions with the lowest intensities. In these areas, the contribution of neutrons from the adjacent wavelength spectrum are contributing most significantly. This results in the artifact manifesting itself as a steep drop at the short wavelength end in the retrieved transmission spectrum.

### Reference sample results

**Figure 4:**
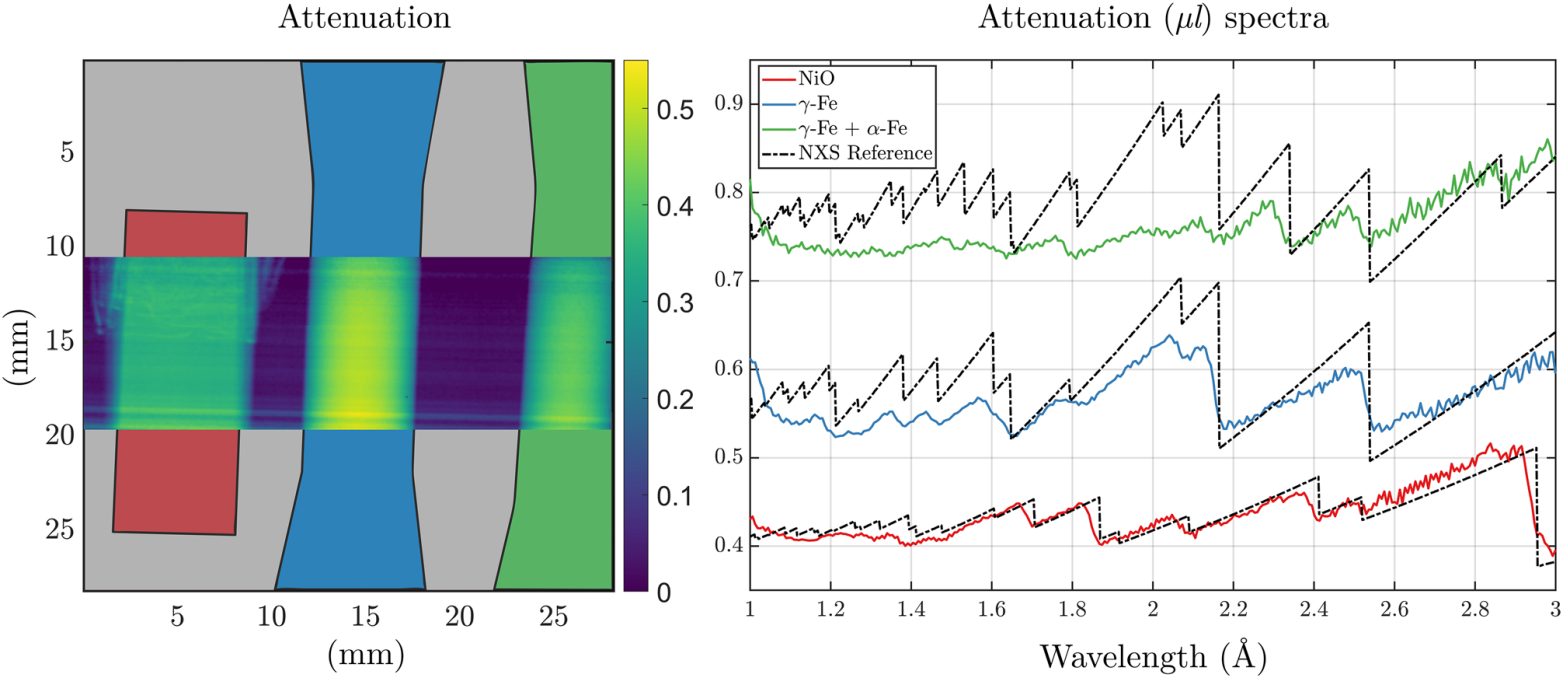
Left: White beam attenuation contrast images, integrated over all ToF bins, of the NiO anode plate (red) and of the two dogbone-shaped stainless steel samples in virgin austenitic (FCC) state (blue) and after tensile deformation and partial martensitic phase transition (green). Right: Bragg edge transmission spectra of the respective samples, as revealed by the FOBI method and as a reference nominal corresponding spectrum for Fe and NiO calculated with the NXS^29^ software (black dash-dotted). Note that the vertical axis is in arbitrary units to avoid overlap between the spectra of the different materials in the figure.

In order to show the method’s ability for Bragg edge imaging we measured a series of polycrystalline samples. First, we probed the Bragg edge transmission spectra of three different samples in a first single exposure. The three samples were the nickel oxide anode plate of a solid oxide fuel cell, and the two dogbone samples of SLM-processed 304, one in the as-built condition and one after being subjected to uniaxial deformation to 52$\%$ nominal strain. The latter resulted in a homogeneous deformation and thus a partial deformation-induced martensitic transformation. Figure 4 shows a sketch of the sample shapes and their full spectrum attenuation contrast images within the limited beam cross section. Next to the image, the respective retrieved attenuation spectra together with corresponding reference attenuation spectra are shown separately for each sample in arbitrary units. The reference spectrum of the nickel oxide compound were calculated using the NXS^29^ software and the crystallographic properties taken from^30^. The reference spectra for the two dogbone samples were calculated for pure austenite and for a phase fraction of $f_M = 40\%$ of BCC martensite. It is observed that the most significant Bragg edges of the nickel oxide and austenite samples are retrieved correctly. Significant deviations, on the longer wavelength side are considered to be due to microstructure and alloying elements of 304L steel, while towards the very end beyond 2.8 $\AA$ the spectral overlap is playing a role, as discussed earlier. The deviations on the shorter wavelength side can be ascribed to the deteriorating wavelength resolution. The deformed steel sample on the other hand displays, as expected, the appearance of the (211) Bragg edge at approximately 2.3 $\AA$, corresponding to the martensitic BCC phase. However, the attenuation spectrum below these wavelengths looks deprived of other nominal Bragg edges. This may partially be due to the formation of crystallographic texture due to deformation, also visible in the apparent peak broadening at longer wavelengths. However, it appears to be mainly due, again, to the decrease of wavelength resolution together with an increasing number of Bragg edges in close vicinity to each other.

**Figure 5:**
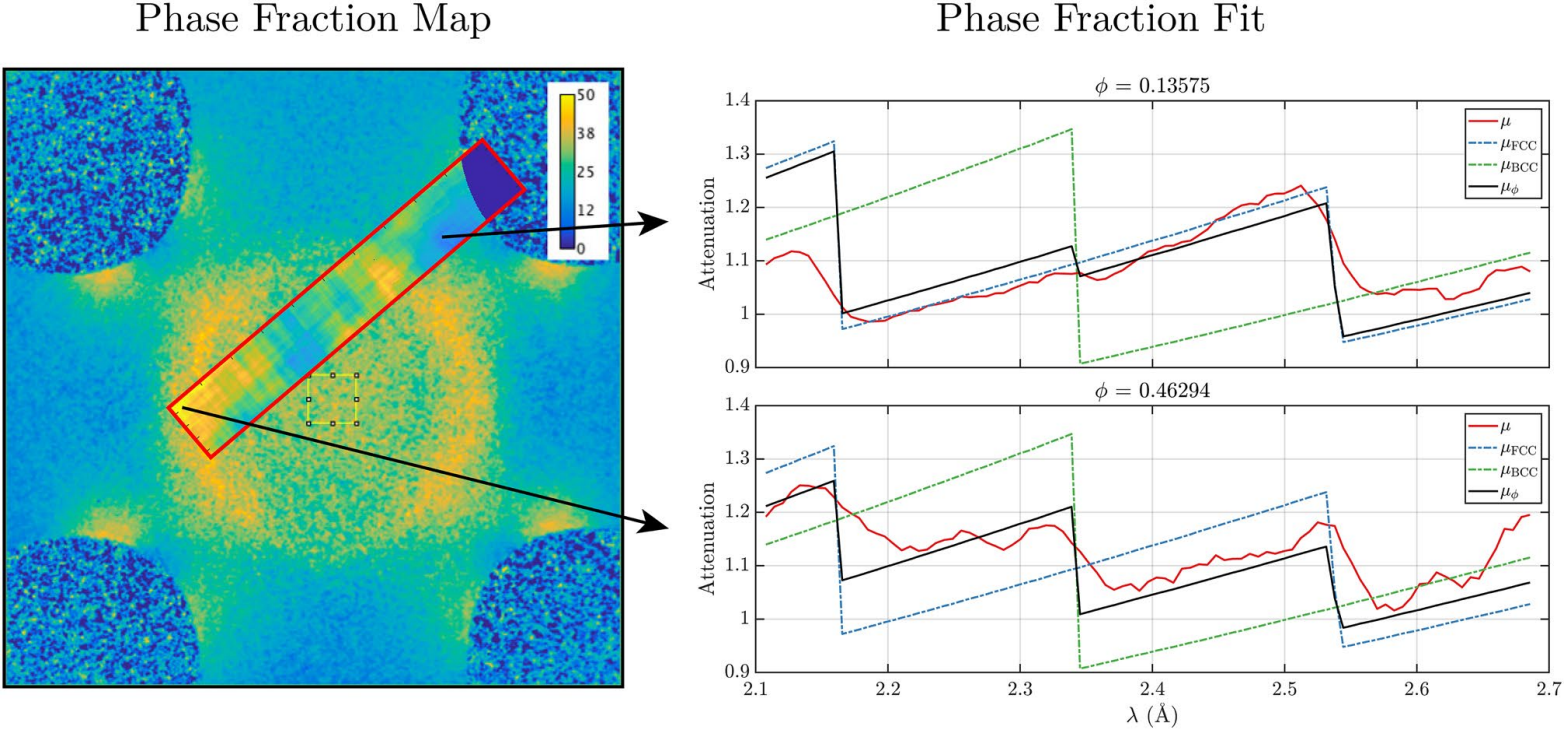
Left: Phase fraction map of the cruciform sample measured with a continuous cold neutron beam with a double crystal monochromator^5^ with an inset of the phase fraction map obtained with the FOBI measurement at the corresponding probed area. Right: Attenuation spectra of single pixels of the FOBI phase map at two positions: (top) in an area of low martensitic phase fraction and (bottom) where a high fraction of martensite was measured. The attenuation spectra include the measurement (red) and the NXS reference for the FCC (blue dot-dashed) and BCC (green dot-dashed) structures as well as their combination according to the fitted phase fraction $f_M$ (black).

Finally, the cruciform sample was analyzed. The initially fully austenitic (FCC) sample that has been subjected to a $90^\circ$ LPC displays a distinct pattern of stress concentrations with high martensitic (BCC) phase fractions. These have previously been mapped with Bragg edge neutron imaging with a large field of view at PSI^5^. In order to demonstrate the Bragg edge imaging capability of the FOBI method we analyzed the FOBI data with respect to the local phase fraction. Note that the FOBI data was taken in 4h while the phase map utilizing a continuous beam was recorded with a total exposure time of one day. In addition, the latter could exploit the significantly higher contrast at the longer wavelength Bragg edges around 4 $\AA$. With the emerging ferritic martensitic phase new Bragg edges appear, accompanied by a loss of contrast of the former austenitic ones. The phase fraction, $f_M$, of the new structural phase has been evaluated by minimizing the squared difference between the measured attenuation spectrum, $\mu (\lambda )$, and a linear combination of the nominal attenuation spectra of the two phases according to:

9 $$\begin{aligned} f_M = \mathop {\text {arg min}}\limits _{f_M} \sum _\lambda |\mu (\lambda )-\left[ f_M\mu _{\text {BCC}}(\lambda )+(1-f_M)\mu _{\text {FCC}}(\lambda )\right] |^2. \end{aligned}$$

The equation was applied only for the wavelength range from 2.1 $\AA$ to 2.7 $\AA$, where the wavelength resolution is best and where the differences between the two structural phases are largest. Figure 5 shows the phase fraction map of the cruciform sample obtained in previous measurements reported elsewhere^5^ with an overlay of the result of the FOBI method in the limited region homogeneously exposed in the POLDI beam, with its limited beam cross section. The figure also shows two examples of how the phase fraction is fitted from the measured spectrum in individual pixels. The strong (211) Bragg edge at 2.3 $\AA$ corresponding to the martensitic BCC structure appears weak and almost negligible where the phase fraction is fitted to be about 13% (Fig. 5 right top) whereas it becomes distinguished where the phase fraction is fitted to be 46% (Fig. 5 right bottom). Despite the smaller field of view, we find a good match between the phase fractions obtained using the FOBI method and the reference measurement. Both the spatial localization and quantification of the sample areas with a significant increase of martensitic phase due to strain concentrations due to biaxial loading are found to be in good agreement with the reference measurement and thus also with finite element simulations of induced stresses, diffraction measurements and EBSD samples^31^.

### Figure of merit

**Figure 6:**
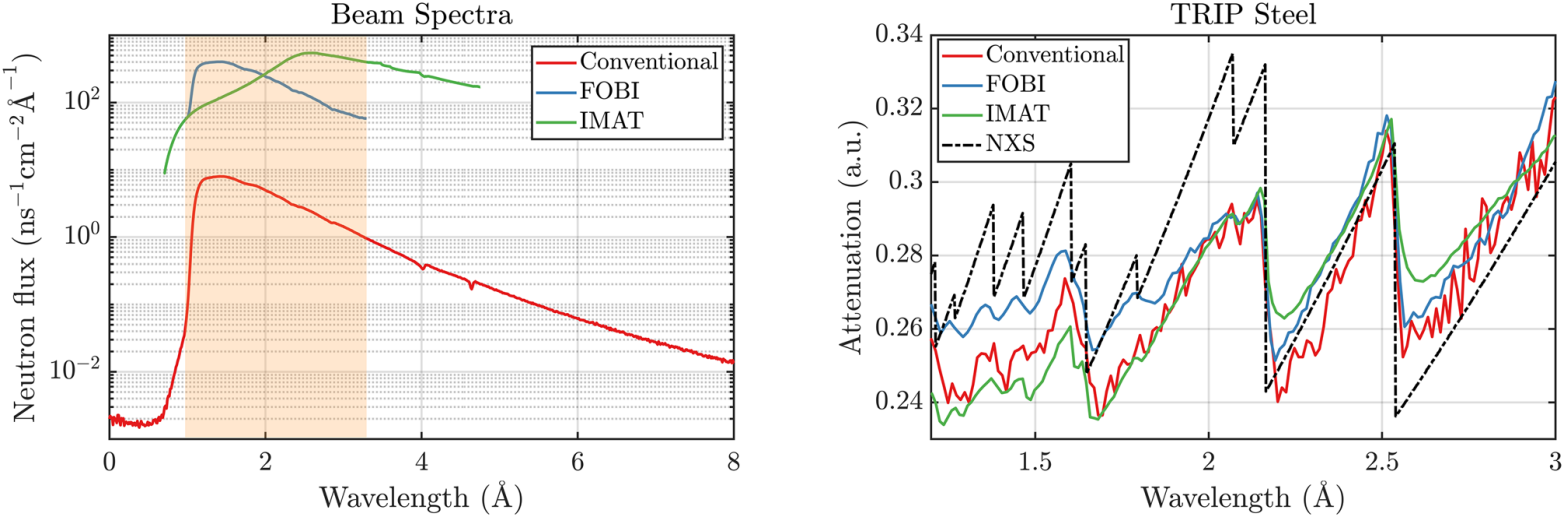
Left: Measured beam spectra in neutron flux unit for the conventional single slit chopper (red) and the FOBI method (blue) at the POLDI beamline, and at the IMAT beamline (green). The light red shaded area marks the wavelength range that is achieved with the FOBI technique. Right: Attenuation spectra of the TRIP steel cruciform sample for the three respective techniques and the theoretical reference calculated with the NXS^29^ software.

The performance of the FOBI method has been benchmarked with the respective conventional chopper based ToF method at our continuous neutron source using a single slit chopper, as well as with a separate measurement carried out at the IMAT^32^ beamline. Figure 6 shows the neutron flux $\left( \text {n}\text {s}^{-1} \text {cm}^{-2}\right)$ and the attenuation spectrum of a TRIP steel sample, measured for the same total exposure time of 4 hours with the conventional chopper at POLDI, with the novel FOBI method and at the imaging and diffraction instrument IMAT at target station 2 of the ISIS pulsed neutron source of the Rutherford Appleton Laboratory. It is observed that the neutron flux incident on the sample is increased significantly when using the FOBI technique, reaching the same order of magnitude as at the IMAT instrument. However, it must be noted that IMAT features a cold neutron spectrum while POLDI is a thermal neutron beamline with intensity distributions peaking at different wavelengths of about 2.6 $\AA$ and 1.4 $\AA$, respectively. The comparison with the single chopper, however, is somewhat biased by the fact that the frequency is limited to less than 1/4 the repetition time of the FOBI pattern. The Bragg edges are retrieved correctly using the FOBI method, as has been sown above as well. An overall reduced noise level with respect to the conventional chopper method is observed, which is consistent with the superior neutron flux. Moreover, the Bragg edge shapes are better depicted by the chopper methods with their sharp symmetric pulse shape compared to the pulsed neutron source featuring the characteristic tail of the pulse reflected on the longer wavelength side of each Bragg edge^6^. The superior flux of IMAT above 2 $\AA$ results in the smoothest spectrum in this region, while the situation reverses below 2 $\AA$, in accordance with the flux comparison in the left side panel of Fig. 6. However, the superior wavelength resolution of IMAT in this spectral region is reflected by sharper edges, even though, both curves with lower flux in this wavelength range, IMAT and the conventional chopper, depart significantly more from the reference curve in this region. This effect still lacks a straightforward explanation, but may be because other (than coherent) scattering contributions were omitted in the calculation of the reference spectra. The reference spectrum was calculated with the NXS software for pure FCC iron, which is the main phase composing the used steel sample. Mismatches in the amplitude of some of the Bragg edges are in general due to microstructure and alloying elements, which have not been accounted for in the approximation of the reference.

Table 1 resumes a quantitative comparison of the different techniques, in terms of instrumental wavelength resolution ($\Delta \lambda /\lambda$), neutron flux and a corresponding FOM. We define the FOM as flux normalized by wavelength resolution relative to the corresponding value for the conventional chopper measurement at POLDI. We provide the flux, resolution and FOM integrated over the wavelength range from 0.96 to 3.28 $\AA$, and the latter at the specific wavelengths of 1.25 $\AA$ and 2.53 $\AA$. The FOBI approach shows a significant increase in performance when compared to the ToF technique at the same source, both in the highlighted bandwidth and for the two separate Bragg edge wavelengths. Despite a mere comparison of the duty cycles corresponding to the FOBI and conventional technique would predict a FOM of 39, we find an overall FOM of approximately 50 for the FOBI method, because the conventional chopper was smaller in diameter. In addition, as mentioned earlier, the conventional chopper was not optimized to the same wavelength band. The nominal flux gain at more comparable conditions would however still amount to a factor of eight for the current case with eight pseudorandom slits in the FOBI chopper. However, we clearly show that the FOBI method can boost the neutron flux of a continuous source ToF measurement by almost an order of magnitude. Further it is shown, that the FOM is comparable for the pulsed source instrument IMAT, with the given bias that a cold neutron instrument (IMAT) is compared to a thermal neutron instrument (POLDI) on the thermal side of the spectrum. Therefore, at certain wavelengths such as at 1.25 $\AA$ the FOM of FOBI equals that of IMAT, while for longer wavelengths IMAT starts getting superior, indicating that the FOBI technique is optimized to operate in specific bandwidths that depend on the source spectrum distribution and instrumental parameters.

**Table 1:** Wavelength resolution ($\Delta \lambda /\lambda$), neutron flux at the 2.53 $\AA$ Bragg edge of austenite and the FOM gain weighted for improvement in the resolution, for the conventional technique using a single slit chopper, the hereby presented FOBI technique and a measurement at the IMAT beamline.

Technique	Neutron flux $\left( \text {n}\text {s}^{-1} \text {cm}^{-2}\right)$	FOM	Resolution	FOM	Resolution	FOM
			@ 1.25 $\AA$	@ 1.25 $\AA$	@ 2.53 $\AA$	@ 2.53 $\AA$
Conventional	9.36$\times 10^3$	1	1.2%	1	0.6%	1
FOBI	6.06$\times 10^5$	51.6	1.2%	51.9	0.6%	49.9
IMAT	7.57 $\times 10^5$	80.9	0.3%^25^	50.8	0.9%^25^	140.1

## Conclusion

We have presented a novel highly efficient ToF method for Bragg edge neutron imaging at a continuous neutron source, which takes advantage of pulse overlap. Corresponding signal processing algorithms are presented that allow such pulse overlap spectra to be analyzed and thus to increase the chopper duty cycle significantly, as compared to conventional implementations. We demonstrate a gain factor of approximately 50 as compared to an ad-hoc conventional chopper solution, which supports a gain factor with respect to an optimized conventional ToF solution of nearly one order of magnitude. We further demonstrate that such efficient implementation of ToF imaging can provide high wavelength resolution Bragg edge imaging capabilities at continuous neutron sources, comparable to those at advanced pulsed spallation sources. However, to achieve the full merit of our method a realization at a cold neutron beamline is required in order to profit from superior Bragg edge contrast in the corresponding wavelength range.

The algorithm for the data processing, which currently is based purely on fast Fourier transforms and simple algebraic operations, has near real-time execution allowing for on the fly data processing. Experimentally, the major concern in the quality of the measured data is represented by the readout gaps of the used detector technology, which affect the signal and hence, the accuracy of the retrieval. However, with the advent of Timepix 3 readout chips the readout gap will become obsolete, enabling a direct measurement of continuous spectra and hence, allowing exclusion or at least significant improvement of a merging procedure.

We have demonstrated the feasibility of the method through measurements of several well-characterized reference samples serving our proof-of-principle. However, it must be regarded that neither the spectrum, the beam (e.g. cross section, collimation) nor the chopper itself, and its slit pattern, have been optimized for the kind of studies we have demonstrated. The results are nevertheless satisfactory and well suited to demonstrate the advance of our approach for high-resolution diffraction contrast imaging at continuous neutron sources thus, fully supporting the implementation of optimized FOBI systems at cold neutron imaging beamlines like e.g. the ICON^33^ instrument at the Paul Scherrer Institute.

## References

[1] Woracek R, Santisteban J, Fedrigo A, Strobl M (2018). Diffraction in neutron imaging-a review. Nucl. Instrum. Methods Phys. Res. A.

[2] Steuwer A, Withers P, Santisteban J, Edwards L (2005). Using pulsed neutron transmission for crystalline phase imaging and analysis. J. Appl. Phys..

[3] Woracek R (2014). 3D Mapping of crystallographic phase distribution using energy-selective neutron tomography. Adv. Mater..

[4] Makowska MG (2017). Coupling between creep and redox behavior in nickel-yttria stabilized zirconia observed in-situ by monochromatic neutron imaging. J. Power Sources.

[5] Polatidis E (2020). Neutron diffraction and diffraction contrast imaging for mapping the trip effect under load path change. Materials.

[6] Santisteban JR (2002). Strain imaging by Bragg edge neutron transmission. Nucl. Instrum. Methods Phys. Res. A.

[7] Strobl M (2012). Time-of-flight neutron imaging for spatially resolved strain investigations based on Bragg edge transmission at a reactor source. Nucl. Instrum. Methods Phys. Res. A.

[8] Morgano M (2020). Investigation of the effect of Laser Shock Peening in Additively Manufactured samples through Bragg Edge Neutron Imaging. Addit. Manuf..

[9] Malamud F (2014). Texture analysis with a time-of-flight neutron strain scanner. J. Appl. Crystallogr..

[10] Treimer W, Strobl M, Kardjilov N, Hilger A, Manke I (2006). Wavelength tunable device for neutron radiography and tomography. Appl. Phys. Lett..

[11] Peetermans S, Grazzi F, Salvemini F, Lehmann E (2013). Spectral characterization of a velocity selector type monochromator for energy-selective neutron imaging. Phys. Procedia.

[12] Tremsin A (2012). High-resolution strain mapping through time-of-flight neutron transmission diffraction with a microchannel plate neutron counting detector. Strain.

[13] Iwase K (2012). In situ lattice strain mapping during tensile loading using the neutron transmission and diffraction methods. J. Appl. Crystallogr..

[14] Makowska MG (2016). In situ time-of-flight neutron imaging of NiO-YSZ anode support reduction under influence of stress. J. Appl. Crystallogr..

[15] Dabah E (2017). Time-resolved Bragg-edge neutron radiography for observing martensitic phase transformation from austenitized super martensitic steel. J. Mater. Sci..

[16] Strobl M (2011). Time-of-flight neutron imaging at a continuous source: proof of principle using a scintillator CCD imaging detector. Nucl. Instrum. Methods Phys. Res. A.

[17] Strobl M (2009). Future prospects of imaging at spallation neutron sources. Nucl. Instrum. Methods Phys. Res. A.

[18] Xiao Z (2011). Coded source neutron imaging at the PULSTAR reactor. Nucl. Instrum. Methods Phys. Res. A.

[19] Fenimore EE (1978). Coded aperture imaging: predicted performance of uniformly redundant arrays. Appl. Opt..

[20] Damato, A. L. & Lanza, R. C. Coded source imaging for neutrons. In*8th World Conference on Neutron Radiography, WCNR-8* 165–173 (2008).

[21] Zou Y (2011). Coded source neutron imaging with a MURA mask. Nucl. Instrum. Methods Phys. Res. A.

[22] Stuhr U (2005). Time-of-flight diffraction with multiple frame overlap Part II: the strain scanner POLDI at PSI. Nucl. Instrum. Methods Phys. Res. A.

[23] Lyon D (2010). The discrete fourier transform, part 6: cross-correlation. J. Object Technol..

[24] Wiener N (1950). Extrapolation, Interpolation, and Smoothing of Stationary Time series: with engineering applications.

[25] Ramadhan RS (2019). Characterization and application of Bragg-edge transmission imaging for strain measurement and crystallographic analysis on the IMAT beamline. J. Appl. Crystallogr..

[26] Tremsin A (2011). High resolution Bragg edge transmission spectroscopy at pulsed neutron sources: proof of principle experiments with a neutron counting MCP detector. Nucl. Instrum. Methods Phys. Res. A.

[27] Tremsin A, Vallerga J, McPhate J, Siegmund O (2014). Optimization of Timepix count rate capabilities for the applications with a periodic input signal. J. Instrum..

[28] Polatidis E, Čapek J, Arabi-Hashemi A, Leinenbach C, Strobl M (2020). High ductility and transformation-induced-plasticity in metastable stainless steel processed by selective laser melting with low power. Scr. Mater..

[29] Boin M (2012). NXS: a program library for neutron cross section calculations. J. Appl. Crystallog..

[30] NiO Crystal Structure: Datasheet from “PAULING FILE Multinaries Edition – 2012” in SpringerMaterials (https://materials.springer.com/isp/crystallographic/docs/sd_0557109). Copyright 2016 Springer-Verlag Berlin Heidelberg & Material Phases Data System (MPDS), Switzerland & National Institute for Materials Science (NIMS), Japan.

[31] Polatidis E (2018). Suppressed martensitic transformation under biaxial loading in low stacking fault energy metastable austenitic steels. Scr. Mater..

[32] Kockelmann W (2018). Time-of-flight neutron imaging on IMAT@ISIS: a new user facility for materials science. J. Imaging.

[33] Kaestner A (2011). The ICON beamline-a facility for cold neutron imaging at SINQ. Nucl. Instrum. Methods Phys. Res. A.

